# Operationalizing atypical gaze in toddlers with autism spectrum disorders: a cohesion-based approach

**DOI:** 10.1186/s13229-018-0211-y

**Published:** 2018-04-10

**Authors:** Quan Wang, Daniel J. Campbell, Suzanne L. Macari, Katarzyna Chawarska, Frederick Shic

**Affiliations:** 1 Child Study Center, Yale School of Medicine, 40 Temple St Suite 7D, New Haven, CT 06510 USA; 2 Vertex Pharmaceuticals Incorporated, 50 Northern Ave, Boston, MA 02210 USA; 3 Center for Child Health, Behavior and Development, Seattle Children’s Research Institute, 2001 8th Ave Suite 400, Seattle, WA 98121 USA; 4 Department of Pediatrics, University of Washington, Seattle, WA USA

**Keywords:** Autism, ASD, Eye tracking, Cohesion, Visual attention, Attentional synchrony, Atypicality

## Abstract

**Background:**

Multiple eye-tracking studies have highlighted the “atypical” nature of social attention in autism. However, it is unclear how “atypical” or “typical” should be quantified.

**Methods:**

We developed a method for identifying moments when members of a group looked at similar places (High-Cohesion Time Frames; HCTFs). We defined typicality as the proximity of gaze points to typically developing (TD) gaze points during TD HCTFs. Comparing toddlers with ASD (*n =* 112) to developmentally delayed (DD, *n =* 36) and TD (*n =* 163) toddlers during a video with Dyadic Bid, Sandwich-Making, Joint Attention, and Animated Toys conditions, we examined (a) individual typicality scores, (b) the relationship between typicality and symptom severity, and (c) HCTF distributions associated with each diagnostic group.

**Results:**

The ASD group had lower gaze typicality scores compared to the TD and DD groups in the Dyadic Bid and Sandwich-Making conditions but not during Animated Toys. The DD and TD groups did not differ in any condition. Correlational analyses indicated that higher typicality scores were associated with increased looking at pre-planned locations of the scene indexed by each experimental condition. In the ASD group, lower gaze typicality was associated with more severe autism symptoms. Examining ASD HCTFs, the gaze of toddlers with ASD was least cohesive during Dyadic Bid and most cohesive during Animated Toys.

**Conclusion:**

In contrast to non-ASD groups, toddlers with ASD show high cohesion during salient nonsocial events, suggesting that consistency in looking strategies may depend more on perceptual features. These findings are consequential for understanding individual differences in visual attention in ASD and for the design of more sensitive biomarker tasks for stratification, between-group differentiation, and measuring response to treatment.

**Electronic supplementary material:**

The online version of this article (10.1186/s13229-018-0211-y) contains supplementary material, which is available to authorized users.

## Background

Eye tracking has been widely used to study gaze behaviors and visual attention and cognition in individuals with and without autism spectrum disorder (ASD) [1, 2]. The most prevalent approach to parsing gaze behaviors involves identifying a priori regions of interest (ROIs) in a displayed scene (e.g., faces, hands, background) and analyzing gaze behaviors as they relate to these ROIs (e.g., how looking times at ROIs differ across populations and experimental conditions). Studies employing ROI approaches have demonstrated that, compared to controls, toddlers with ASD spend less time attending to people, their faces, and their goal-oriented activities [3–7]; for a recent meta-analysis of eye tracking in autism research see [1, 8]. These studies also highlight the differential impact of social context on attention in toddlers with ASD compared to those without autism. For instance, unlike typically developing (TD) and developmentally delayed (DD) toddlers, toddlers with ASD show decreased attention to a speaker’s face only when the person looks at or speaks to the viewer and not in other conditions [3]. In addition, in children with ASD, heterogeneous gaze patterns in response to dynamic social stimuli have been linked to differences in the severity of autism symptoms and levels of developmental functioning as measured 1–2 years later [9].

These findings demonstrate how context and development affect the gaze behaviors of children with ASD, highlighting the complexities of precisely defining atypical gaze behavior. Furthermore, “atypical” can only be defined in reference to “typical,” and defining “typical” behavior poses some challenges. For instance, gaze patterns vary developmentally, with attention to faces gradually increasing during the first year of life [10–13] and the significance of looking at the eyes or mouth changing as children begin to acquire language [14]. Even within normative samples studied within narrowly defined developmental periods, there is high inter-individual variability in gaze behaviors. For instance, Tenenbaum and colleagues [15] demonstrated large inter-individual variation in looking preferences for the mouth of a speaking or smiling face in young, TD infants. Similar variability has been linked to language outcomes in infant siblings of children with ASD [16]. These results illustrate that using a single norm (or simple set of ROIs) as representative of typical gaze patterns might not reflect the complex realities of intergroup or contextual gaze dynamics. These complexities are further compounded in studies of videos in which the contextual changes vary in a moment-by-moment fashion alongside corresponding ROIs. Moreover, ROI-based approaches to gaze analyses in ASD are based on top-down (investigator-defined) strategies, and differences in how ROIs are defined may introduce discrepancies when comparing results across different studies. Finally, it is not clear if ROIs, defined by experimenters who themselves are typically developing adults, fairly capture axes of variation in atypical or very young populations, especially in response to complex dynamic stimuli, in which context unfolds rapidly along multiple dimensions. These issues surrounding the multiplicity of interpretative possibilities in ROI analyses, at the core, stem from the a priori assumption of spatial points of regard characterizing constructs of interest that are more-arbitrarily defined than they are data-driven (for additional discussions, including alternative algorithms, see [17, 18] and Additional file 1: Materials 1).

Here we propose a new approach to the analysis of dynamic eye-tracking data that is based on empirically derived gaze behaviors of TD children, applicable to studies of scene looking with a priori ROI hypotheses as well as to those without. Despite the observed inter-individual variability in scanning patterns among TD children, attributable to individual neural, biological, and experiential differences, there are moments in time when the gaze behaviors of TD children converge on the same spatial location. This convergence suggests a common response to a combination of perceptual and semantic scene characteristics. We propose to use the term *cohesion* to describe this phenomenon of convergence by multiple individuals on the same area of the visual scene within a specified time frame, i.e., when the gaze points of individuals fall within close proximity to one another and those individuals participate in a consistent, unified visual experience. Each frame for each individual can be assigned a typicality score in reference to normative patterns of cohesion derived from the TD group. Using a cohesion value metric, we identified frames where the cohesion of gaze behaviors within the typical group was the highest (high-cohesion time frames, HCTFs) and argue that the analysis of HCTFs can inform studies of gaze behaviors across typical and atypical development in novel and generative ways. By identifying when and where the gaze behaviors of TD children converge in response to complex visual scenes, we can define spatial and temporal windows reflective of typical gaze behavior patterns. Subsequently, we can compute indices of similarity between TD and atypically developing samples during these windows to quantify the degree of deviation of gaze behaviors from those typically observed. Similarly, we can examine what constitutes the most “consistent” gaze behaviors *within* the notoriously heterogeneous samples of children with developmental issues by computing cohesion indices specific to these samples. That is, rather than examining deviance from a norm based on TD samples, we can also establish “norms” for specific clinical groups.

In the present study, we applied the cohesion approach to eye-tracking data derived from a large sample of toddlers with autism and developmental delays, as well as typically developing controls. We aimed to operationalize and examine gaze behaviors in clinical groups by building a data-driven normative model of gaze behavior in TD toddlers, comparing the performance of the ASD, DD, and TD groups using a gaze typicality score within the context of this normative model, and examining the relationship between gaze typicality scores and autism symptoms in the ASD group. We also aimed to investigate “normative” gaze patterns within the ASD and DD groups by examining how the proportion of HCTFs differed across conditions in ASD and DD toddlers, as compared to TD toddlers.

## Methods

### Participants

Participants included toddlers with ASD (age *M* = 22.39, SD = 3.02 months, *n =* 112), DD (age *M* = 21.71, SD = 3.38 months, *n =* 36), and TD (age *M* = 21.89, SD = 3.39 months, *n =* 163). ASD participants were recruited at a university-based research clinic specializing in the early differential diagnosis of autism and other developmental disorders. The study of children with ASD at this early age afforded the examination of visual gaze strategies typically at the age of first diagnosis and therefore before the potential secondary effects of interventions would likely take hold. The TD and DD toddlers had no family history of autism in first or second degree relatives. Developmental skills were evaluated using Mullen Scales of Early Learning (MSEL, 1995 [19]); (see Table 1). The MSEL captures developmental functioning in nonverbal (fine motor and visual reception) and verbal (receptive language and expressive language) domains. For this study, developmental quotients were computed for the verbal (VDQ) and nonverbal (NVDQ) scores. The severity of autism symptoms was measured using the Autism Diagnostic Observation Schedule-Generic Module 1 (ADOS-G [20, 21]); (see Table 1). The ADOS-G provides scores in the domains of social affect (SA) and restrictive and repetitive behaviors (RRB), as well as a total score reflecting the sum of SA and RRB scores. The three groups did not differ with regard to age (*F*(2, 308) = 1.00, *p* = .37). The ASD group consisted of 85.7% males, as compared to 88.9% in DD and 59.5% in TD groups (*χ*^2^(2) = 28.4, *p* < .01). The ASD and DD groups were comparable with regard to MSEL NVDQ (*p* = 0.25), and both had lower scores than the TD group (*p*s < .001). The MSEL VDQ of the ASD group (*M* = 55.8, SD = 2.4) was significantly lower than that of the TD (*p* < .001) and DD groups (*p* < .01). The VDQ of the DD group was also lower than that of the TD group (*p* < .001). All ASD diagnoses were based on clinical best estimate (CBE). In 79.5% (*n* = 89) of cases, CBE was conducted in a follow-up visit at 36 months (mean age at eye tracking 22.5 months; at CBE 38.7 months); in the remaining 20.5% (*n* = 23) of cases, CBE was conducted at the time of eye tracking (mean age 22.2 months). CBE was based on the direct assessment of developmental, social, communication, and adaptive skills, as well as review of developmental and medical history, by a multidisciplinary team of expert clinicians. Standard measures included the ADOS-G [20, 21], MSEL [19], PLS-5 [22], Vineland [23], and ADI-R [24]. Previous studies have indicated that CBE diagnoses of ASD in clinic-referred children are highly stable (~ 90%) between the second and third year of life [25–27]. Given the large size of our samples, this is unlikely to significantly impact study results. The DD group included toddlers with a score less than 1.5 SDs below age-norms on one or more subscales of the MSEL and included toddlers with global developmental delays or language delays. Children in the TD group exhibited typical developmental profiles. This research was approved by the Yale University Institutional Review Board, and informed consent was obtained from the legal guardians of all participants enrolled in this study. Subsets of this data have been previously reported in [3, 9, 28].

**Table 1:** Sample characterization

	ASD			DD			TD		
Male	85.7%			88.9%			59.5%		
	N	Mean	SD	N	Mean	SD	N	Mean	SD
Age (months)	112	22.39	3.02	36	21.71	3.38	163	21.89	3.39
MSEL NVDQ	110	82.70	16.67	36	85.86	11.81	161	109.96	13.00
MSEL VDQ	110	55.84	30.97	36	69.76	16.89	154	111.34	21.23
ADOS SA	109	13.35	4.66	35	5.83	3.97	–	–	–
ADOS RRB	109	4.07	2.03	35	1.37	1.52	–	–	–
ADOS TOTAL	109	17.42	5.69	35	7.20	4.52	–	–	–

*ASD*, autism spectrum disorder; *DD*, developmental delay; *TD*, typical development; *MSEL*, Mullen Scales of Early Learning; *NV*, non-verbal; *V*, verbal; *DQ*, developmental quotient; *ADOS*, Autism diagnostic observation schedule; *SA*, social affect; *RRB*, restricted and repetitive behaviors

### Stimuli

**Fig. 1:**
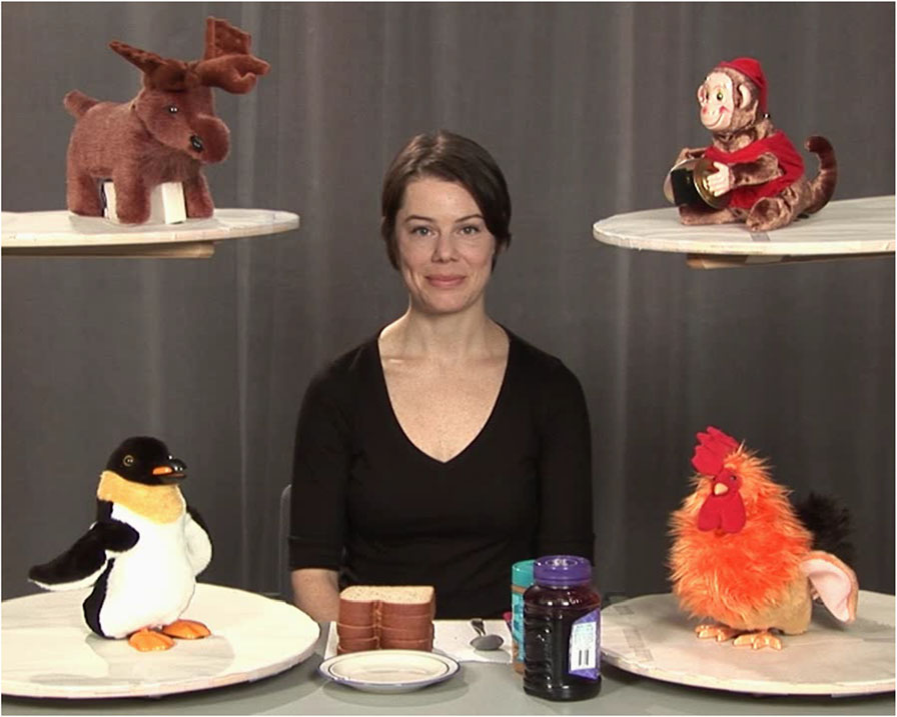
The stimulus shown to participants: a 3-min video depicting an actress engaged in several activities in four interleaved conditions (Dyadic Bid, Sandwich, Joint Attention, and Animated Toys)

The stimulus consisted of a 3-min video depicting an actress engaged in several activities in a setting shown in Fig. 1 (for a detailed description, see [3]). The video has four interleaved conditions (Dyadic Bid, Sandwich, Joint Attention, and Animated Toys), without breaks to re-engage or re-center the child’s visual attention. In the Dyadic Bid condition, the actress looks directly at the camera and uses child-directed speech (e.g., “Hi, baby, how are you today?”) to elicit dyadic (face-to-face) attention (11 episodes, total duration of 69 s). In the Sandwich condition, she looks down at the ingredients and tools on a table with no direct gaze or speech (2 episodes, total duration of 63 s). In the Joint Attention condition, the actress looks up briefly at the camera and then says “uh-oh” as she turns toward one of the toys and looks at it for 4 s (4 episodes, total duration of 30 s). In the Animated Toys condition, the actress looks up briefly at the camera, then a toy begins to move and make noise, followed by the actress turning to look at a toy on the opposite side of the animated toy (4 episodes, total duration of 27 s).

#### Apparatus

An SMI iView X RED 60 Hz eye-tracking system was used to record toddlers’ eye movements. Eye-tracking data were post-processed with a custom data pipeline programmed in MATLAB. Processing steps included calibration, recalibration, blink detection [29, 30], and cohesion analysis. Participants were included if they spent more than 30% of the video attending to the scenes (i.e., if the amount of time the eye tracker detected looking at the monitor, divided by the total stimulus presentation time, was greater than 30%) and had calibration uncertainty less than 2 degrees.

#### Procedure

The eye-tracking experiment was conducted in a dark and quiet room. A toddler sat in front of a 24-in. computer screen at an average distance of 75 cm. The experiment began with a child-friendly video to direct the toddler’s attention to the screen, followed by a five-point calibration before the stimulus video began. Calibration targets included dynamic animations with sound (e.g., a walking cartoon tiger with a meowing sound).

### Analytic strategy

#### Normative mode

**Fig. 2:**
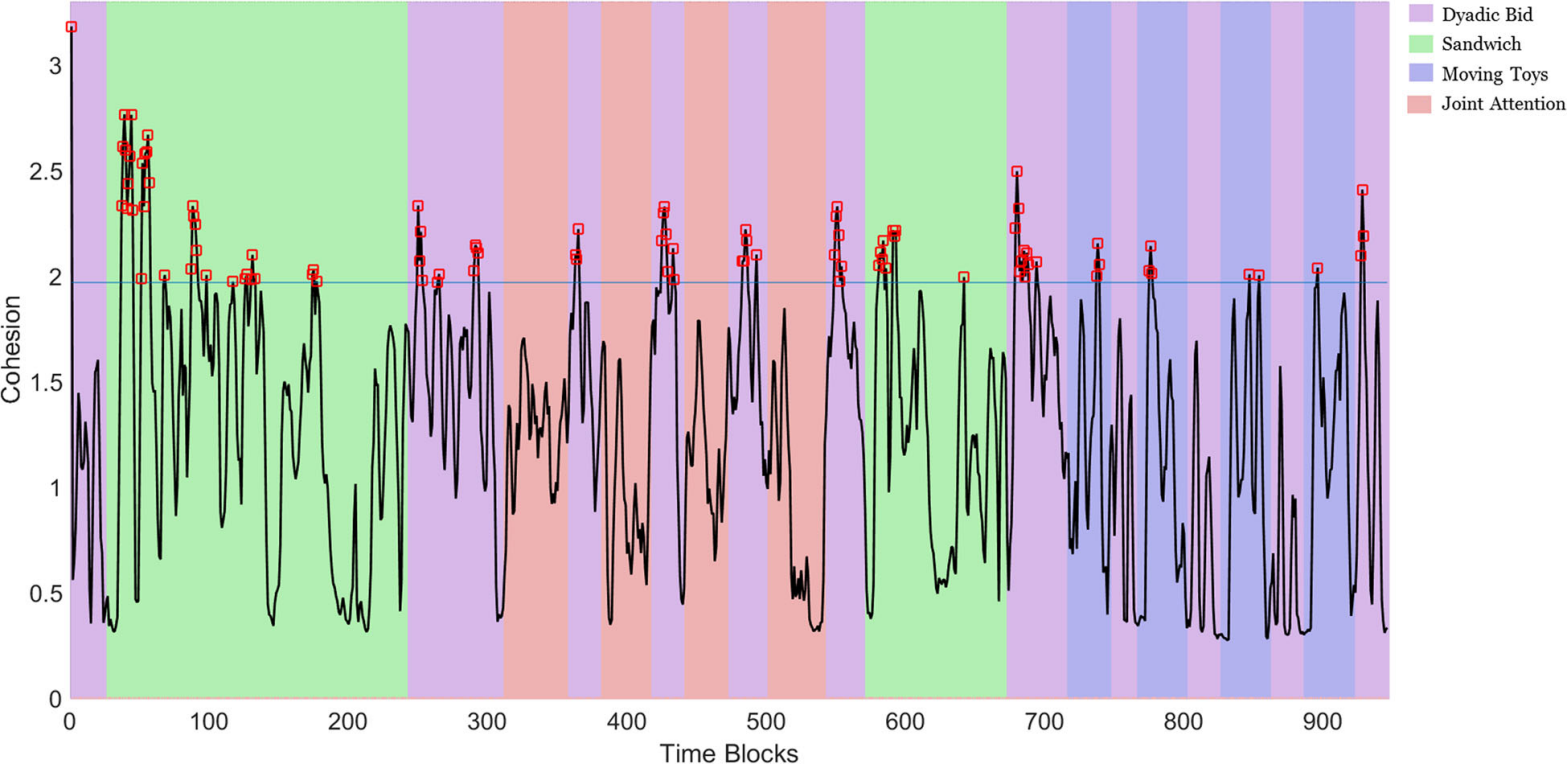
High-cohesion time frames (HCTFs) in the normative model. We calculated the median cohesion value among TD toddlers for each time frame, shown as the black line plot. HCTFs included in the normative model are indicated with red boxes. Time frames belonging to one of four different conditions are identified by corresponding background colors: Purple: Dyadic Bid; light green: Sandwich; Blue: Animated Toys; Orange: Joint Attention. Typicality scores in different conditions represent the median of cohesion values within the HCTFs in the corresponding video condition

In order to operationalize typical scanning patterns, we created a normative model using the following steps (see Additional file 1 Materials 1 for more details): step 1 aimed to define a *cohesion value* which would represent the similarity in gaze locations during a given time frame (each 200 ms) between a TD toddler and all other TD toddlers (i.e., how similar a TD participant was to other TD participants). More formally, the cohesion value was defined as being proportional to the inverse median pairwise distance between a participant and all other participants in his or her group. In step 2, we defined HCTF as the time frames when the median cohesion values of TD participants were the top 10% of all frames. Conceptually, an HCTF represents a time interval when TD toddlers focus their attention on a similar location of the screen (i.e., when a majority of TD participants are looking at the scene content in a similar way). In step 3, we defined *typicality scores* as cohesion values during HCTFs, representing the similarity of each participant’s gaze patterns to TD participants during moments when the TD group exhibited the most cohesive gaze behavior. Typicality scores were calculated for each individual, for each condition (for an example of time-varying cohesion values across conditions, see Fig. 2). We compared typicality scores between diagnostic groups (ASD, DD, and TD), across conditions, using linear mixed models (compound symmetry repeated covariance structure, type III sum of squares), and post hoc comparisons Holm-Bonferroni corrected for multiple comparisons (consistent with our prior work [31]). To clarify how typicality scores corresponded to spatial locations in each experimental condition, we isolated the HCTFs referenced to the TD sample (i.e., the normative model) and applied conventional ROI analyses to each condition. We then conducted a Pearson’s *r* correlation analysis to examine how spatial ROI looking percentages related to typicality scores across all participants. Pearson’s *r* correlation analysis was used to explore relationships between typicality scores and autism-related symptoms in the ASD group.

#### ASD and DD cohesion models

To examine cohesive behaviors within each diagnostic group, we also created *within-group cohesion models* for ASD and DD toddlers. Similar to the normative model of TD toddlers, toddlers with ASD were compared to all other toddlers with ASD, and DD toddlers to all other toddlers with DD. This aim involved computing the *proportion of HCTFs* for each condition for each within-group cohesion model. Allocation of HCTFs across conditions could deviate from chance (10%, the proportion of frames selected as HCTFs), by exceeding (greater-than-chance proportions of HCTFs within a condition) or being lower than chance (lower-than-chance proportions of HCTFs). Statistical analyses for expression levels of cohesion in different conditions across the diagnostic groups allowed us to identify commonalities in attentional salience that may be shared across members of particular groups (e.g., what draws attention most consistently in the ASD group).

## Results

### Normative model

Cohesion values of the TD group are presented as a line plot, with different video conditions represented by different background colors in Fig. 2. The top 10% of cohesion values in the normative model were labeled as HCTFs and are shown as red rectangles in Fig. 2. This 10% cutoff, determined a priori, was found to be equivalent to a median pairwise distance between TD participants of less than 49.8 pixels on the screen (~ 1.5° of visual angle), which is correspondent to the size of foveal avascular zone, the area of highest acuity in the visual field [32, 33]. Interestingly, the algorithm identifying HCTFs within the TD group revealed no HCTFs drawn from the Joint Attention condition. For this reason, subsequent analyses do not contain the Joint Attention condition.

### Typicality score group comparison (see Fig. 3 and Table 2)

To examine whether children in the DD and ASD groups showed similar gaze behaviors to the TD group during periods when TD gaze behaviors were highly convergent, we computed a diagnosis (3) × condition (3) linear mixed model on typicality scores. The analysis indicated a diagnosis effect (*F*(2, 307.6) = 24.9, *p* < .001) and a diagnosis × condition interaction (*F*(4, 613.6) = 5.9, *p* < .001), but no condition effect (*F*(2, 613.4) = 2.1, *p* = .13). The ASD group had lower typicality scores compared to the TD and DD group in Sandwich (*p* < .001, Cohen’s *d* = − 0.64; *p* = .02, *d* = − 0.41, respectively) and Dyadic Bid (*p* < .001, *d* = − 0.93; *p* < .001, *d* = − 0.80) conditions but not Animated Toys (*p* = .06, *d* = − 0.34; *p =* .94, *d* = − 0.02). The DD group did not differ from the TD group in any conditions (Sandwich, *p* = .88; Dyadic Bid, *p* = 1.00; Animated Toys, *p* = .36). Inclusion of NVDQ, VDQ, calibration accuracy, or percentage valid as covariates in the linear mixed model did not change these statistical results (see Additional file 1 Materials 1).

**Fig. 3:**
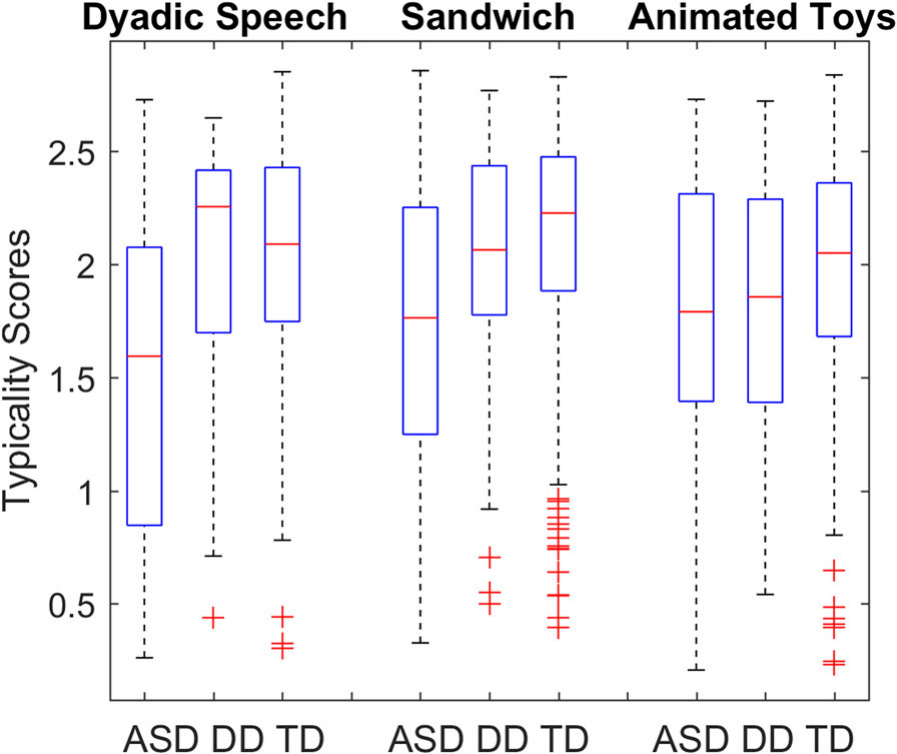
Typicality score box plots for ASD, DD, and TD groups in the normative model. Comparisons are made between the diagnostic groups in the three video conditions. For Sandwich: ASD < (DD*,TD**); Dyadic Bid: ASD < (DD**,TD**); Animated Toys: no differences. *p < .05, **p < .01

**Table 2:** Mean and standard deviation (SD) of typicality scores of the ASD, DD, and TD groups in the Sandwich (SW), Dyadic Bid (DB), and Animated Toys (Toy) conditions. Joint Attention (JA) is not included since, in the TD normative model, no cohesive frames were allocated to the JA condition

Typicality scores	Diagnosis								
	ASD			DD			TD		
Conditions	SW	DB	TOY	SW	DB	TOY	SW	DB	TOY
Mean	1.70	1.47	1.77	1.98	2.01	1.77	2.09	2.03	1.96
SD	0.67	0.71	0.64	0.60	0.55	0.66	0.56	0.52	0.55

### Correlation between typicality scores and ROI looking-time percentages across all groups

ROI looking-time percentages are presented in Additional file 1: Material 1, Table S1. In the Sandwich (SW) condition, typicality scores were positively correlated with looking at the table (the activity area of sandwich making) and negatively correlated with other ROIs. In the Dyadic Bid (DB) condition, typicality scores were positively correlated with looking at the face of the actress and negatively correlated with other ROIs. In the Toy condition, typicality scores were positively correlated with looking at the four toys and negatively correlated with other ROIs (Table 3). Similar relationships were observed when groups were considered independently (see Additional file 1: Material 1, Table S2).

**Table 3:** Correlation between typicality score and percentage looking at predefined regions of interest (ROIs) in the cohesive frames of the normative model, stratified by experimental condition across all groups

Looking percentage on ROIs***	Face	Toys	Body	Table	BG
SW typicality score	− .47	− .46	− .43	**.75**	− .48
DB typicality score	.79	− .51	− .40	− .45	− .51
Toy typicality score	−.24	.68	− .24	− .19	− .57

Bold entries highlight positive associations

### Correlation between typicality score and severity of autism symptoms in the ASD group

Subsequently, we conducted Pearson’s *r* correlation tests between typicality scores for each condition and ADOS social affect (SA) and restricted and repetitive behavior (RRB) scores, as an exploratory analysis. ADOS SA scores were negatively correlated with the typicality scores in the Sandwich (*r*(109) = − 0.235, *p* = .014) and Dyadic Bid condition (*r*(109) = − 0.199, *p* = .038), but not in Animated Toys (*r*(109) = − 0.06, *p* = 0.538). ADOS RRB scores were not correlated with any condition (*r*(109) = − 0.158, 0.003, and − 0.117, all *p*s > .05, for Sandwich, Dyadic Bid, and Animated Toys conditions respectively). Statistical results were unchanged using Spearman’s rank correlation.

### Proportion of frames with highest cohesion scores

For ASD and the DD groups, we identified the proportion of HCTFs in each condition (Table 4). We excluded the Joint Attention condition for comparability with the TD group, which had no HCTFs in the Joint Attention condition in the normative model. Similarly, for comparability, we maintained the 10% threshold for HCTF identification. Because the 10% of frames, across all conditions, with the highest cohesion were defined as HCTFs, if guided purely by random uniform chance, each condition would be expected to show a 10% composition of HCTFs. Thus, conditions with more than 10% of HCTFs were labeled as demonstrating greater-than-chance proportions of HCTFs (i.e., showing more cohesion that would be expected by chance, thus demonstrating more homogeneous group behavior) and conditions with less than 10% of HCTFs were labeled as demonstrating lower-than-chance proportions.

**Table 4:** Mean and standard deviation (SD) of proportion (in %) of high-cohesion time frames (HCTF) in the Sandwich (SW), Dyadic Bid (DB), and Animated Toys (TOY) conditions in the ASD, DD, and TD groups. Above 10% is greater-than-chance proportions of cohesive frames, under 10% is less-than-chance

Typicality scores	Diagnosis								
	ASD			DD			TD		
Conditions	SW	DB	TOY	SW	DB	TOY	SW	DB	TOY
Mean	14.32	6.30	14.60	9.79	14.29	3.82	13.42	12.50	6.74
SD	2.20	1.47	3.61	1.11	1.35	0.82	1.70	1.42	3.40

**Fig. 4:**
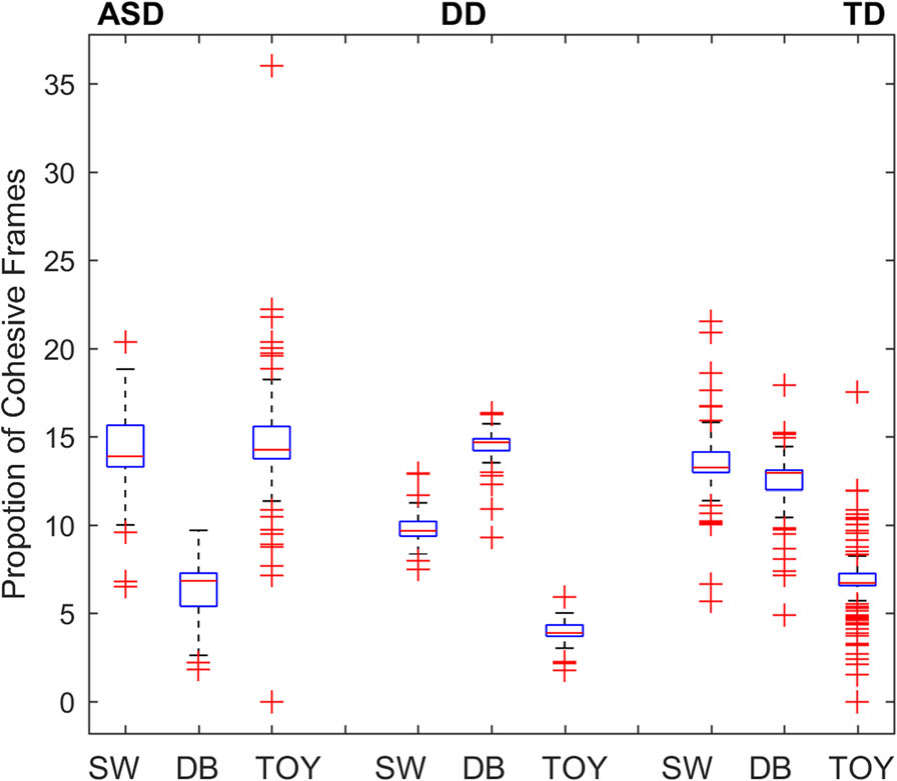
Box plots illustrating proportion of HCTFs in Dyadic Bid (DB), Sandwich (SW), and Animated Toy (TOY) conditions in within-group model. For ASD: DB<SW<TOY; for DD: TOY<SW<DB; and for TD: TOY<DB<SW. All effects *p* < .001

Within-group linear mixed models were run to examine potential differences in the proportion of HCTFs allocated to each condition within each group (Fig. 4 and Table 4). In the TD group, condition was significant (*F*(2, 324) = 532.9, *p* < .001), with post hoc comparisons indicating the highest proportion of HCTFs in the Sandwich condition (13.4%), next highest in the Dyadic Bid condition (12.5%), and the lowest in the Animated Toys condition (6.7%) (all pairwise *p* < .001). In the DD group, condition was significant (*F*(2, 70) = 554.8, *p* < .001), with the highest proportion of HCTFs in the Dyadic Bid (14.3%) condition, next highest in the Sandwich (9.8%) condition, and, like the TD group, the lowest proportion in the Animated Toys condition (3.8%) (all pairwise *p* < .001). In the ASD group, condition was significant (*F*(2, 222) = 272.3, *p* < .001), but, in contrast, the highest proportion of HCTFs occurred in the Animated Toys (14.6%) and Sandwich conditions (14.3%) and the lowest in the Dyadic Bid condition (6.3%) (pairwise *p* < .001, excepting Animated Toys vs Sandwich, *p* = .491). Except for the DD group in the Sandwich condition, all proportions of HCTFs significantly differed from chance (10%) in one sample *T* tests. Between-condition group-specific effect sizes are provided in Additional file 1: Material 1, Table S3.

## Discussion

This is the first study to apply a data-driven cohesion-based approach to the analysis of eye-tracking data collected in response to dynamic complex scenes in toddlers with and without social disability. This study provides a unique perspective on defining atypical gaze behaviors in ASD. We conducted the cohesion model analysis with a sample of over 300 well-characterized toddlers with and without autism and other developmental disabilities at the earliest age of ASD diagnosis. This cohesion approach was data-driven and free of assumptions associated with pre-defined ROIs, though results comparing correlations between typicality and standard ROI-based measures of looking suggested that typicality scores reflected the pre-planned point-of-regard manipulated and targeted by experimental conditions. Differences in patterns of looking in cohesive frames in the normative model replicated results observed in high-level between group analyses in [3] (see Supplement).

Results suggested that the gaze behaviors of toddlers with ASD were most atypical in contexts involving face-to-face interactions (Dyadic Bid) and goal-oriented activities (Sandwich condition). Despite the presence of developmental delays in the DD group, the DD and TD children did not differ in their gaze behaviors in any condition, suggesting that intellectual functioning alone cannot explain the differences observed in the ASD group. These findings are consistent with previous ROI-based findings suggesting atypical gaze behaviors in response to social bids and limited activity monitoring in children with ASD at this age [3, 5].

In the ASD group, lower typicality scores in Sandwich and Dyadic Bid conditions were associated with higher severity of social-affective symptoms. This suggests that cohesion-based metrics may be clinically meaningful and that cohesion may provide a powerful method for indexing severity of autism symptoms and understanding individual variation within the autism spectrum. Given that gaze behavior guides learning throughout development, atypical looking patterns would provide access to different experiences for children with ASD as compared to TD or DD toddlers, potentially leading to more impoverished opportunities for social learning. Our results are consistent with previous work that has suggested that gaze atypicality is associated with symptom severity [9].

There were no differences in gaze patterns during the Animated Toys condition between the ASD and DD groups. This lack of differences could be due to shared similarities in attraction to physical properties of the scene, such as motion [34–37]. It may also be the case that the non-social nature of the Animated Toys condition did not tap into ASD-DD between-group differences in as stark a fashion as the more socially oriented Dyadic Bid and Sandwich conditions. Furthermore, there were no correlations between typicality scores and autism symptoms in the ASD group within the Animated Toys condition, reinforcing the perspective that attention to non-social events may be less powerful for stratification along the autism spectrum.

However, it is important to note that typicality scores only tell us how much toddlers in the ASD or DD groups deviate from TD toddlers; they do not tell us about the inherent gaze patterns within groups, e.g., when and on what areas of the scene children with ASD or DD tend to converge compared to other children within their own group. To address this, we constructed cohesion models for each diagnostic group independently, identifying the moments of highest cohesion within each group, and then examined structural differences in cohesion points across conditions within different diagnostic groups.

In the TD group, toddlers showed greater-than-chance proportions of HCTFs in both Sandwich and Dyadic Bid conditions and lower-than-chance proportions in Animated Toys. This suggests that they were more likely to look at the same region at the same time when potent social cues for attention, such as eye contact and speech or goal-oriented action, were present, but in response to nonsocial events, the TD toddlers showed relatively greater variability. Similarly to the TD group, the DD group showed higher-than-chance proportions of HCTFs in the Dyadic Bid condition and lower-than-chance proportions in Animated Toys. However, unlike the TD group, the proportion of HCTFs during the Sandwich condition in the DD group was at chance level. This finding is consistent with toddlers with developmental delays exhibiting gaze patterns more similar to younger TD toddlers, who spend more time looking at faces, as compared to older TD children, who spend more time looking at hands performing goal-oriented activities [38]. Alternatively, it is possible that variability in gaze patterns in the Sandwich condition in the DD group could be attributed to group heterogeneity in the understanding of daily living skills and activities [5]. By comparison, the ASD group had greater-than-chance proportions of HCTFs in the Animated Toys and Sandwich conditions and, in stark contrast to the TD and DD groups, lower-than-chance proportions of HCTFs in the Dyadic Bid condition. These findings may suggest that within the ASD group, attention was driven more toward perceptually salient nonsocial events.

In the normative cohesion model, one may notice that the TD group had no HCTFs in the Joint Attention condition. Joint attention induces a dynamic process of following the gaze direction of the person on the screen, which is not well-captured by the cohesion model. For example, two participants could both look back and forth between the actress and the target of attention while being locked completely out of phase (one looking at the target when the other is looking at the actress), and thus contribute to low spatial cohesion, despite a similar underlying strategy of attention [39] (see also Additional file 1**:** Material 1, Fig. S2). In addition, Joint Attention is a complex phenomenon that has high variability depending on age, developmental level, and temperamental factors, indexing a skill which is in rapid development during this period [40]. Future work will consider modifications to the cohesion model which can better account for cohesion effects during displays of complex social behavior.

There are several limitations in this study. First, we only applied this method to one video stimulus, and we acknowledge that this approach needs to be validated on additional stimuli and under different experimental contexts. Second, our correlation analysis between typicality score and autism-related symptoms in the ASD group is exploratory and uncorrected for multiple comparisons. Third, for consistency, we applied the same 10% cutoff criterion used for identifying HCTFs in the TD group for all groups but acknowledge that the mapping of 10% to distances/visual angles differs for the ASD (1.9°) or DD (1.6°) groups. Finally, this study involved very young children at the toddler age, and it is not clear whether these methods would be fully applicable across the lifespan. In the future, we hope to employ this technique for other research applications and to further explore the impact of variation in methodological parameters on different participant groups.

## Conclusions

In summary, using our cohesion approach, we identified canonical gaze patterns in response to complex visual scenes and quantified the degree of consistency with which attention is drawn to specific features in the scene within different diagnostic groups. We also evaluated the clinical significance of individual differences from these canonical gaze patterns. Our results showed that this data-driven approach indexed atypical looking in the ASD group compared to the DD and TD control groups in socially charged experimental scenarios. Furthermore, atypical looking patterns during social conditions stratified children with ASD by level of autism symptoms. Finally, our results showed that ASD toddlers as a group exhibited more cohesive behaviors during non-social conditions and less during social conditions—a pattern reversed for DD and TD toddlers. These findings are consequential for understanding individual differences in attention to social targets in toddlers with ASD and for designing more sensitive biomarkers capable of measuring response to treatment.

## Additional file

Additional file 1: Supplementary Information. (DOCX 374 kb)

## Abbreviations

ADOS: Autism diagnostic observation schedule
ASD: Autism spectrum disorder
CBE: Clinical best estimate
DB: Dyadic Bid (video condition)
DD: Developmental delay
DQ: Developmental quotient
HCTF: High-cohesion time frames
JA: Joint Attention (video condition)
MSEL: Mullen Scales of Early Learning
NV: Non-verbal
ROI: Regions of interest
RRB: Restricted and repetitive behaviors
SA: Social affect
SD: Standard deviation
SW: Sandwich (video condition)
TD: Typical development
Toy: Animated Toys (video condition)
V: Verbal
