# Supplementary material for: Operationalizing atypical gaze in toddlers with autism spectrum disorders: a cohesion-based approach
Source: Mol Autism. 2018 Apr 10;9:25. doi: 10.1186/s13229-018-0211-y (PMC5894192; doi:10.1186/s13229-018-0211-y)
Supplement: Supplementary file 1 — Supplementary Information. (DOCX 374 kb) [file 13229_2018_211_MOESM1_ESM.docx]

**Additional file 1: Material 1**

*Introduction*

Alternative approaches to defining regions-of-interest (ROIs) have explored feature-based, bottom-up, biologically-inspired models to predict typical gaze behaviors. In accordance with these models, humans reflexively look at image regions with high visual saliency (1), i.e., regions containing more unique and distinctive visual features (2), including areas of stark contrast and rapid motion. In studies of ASD, bottom-up methods have been applied to adult (3) and child data (4,5). However, computational models based on saliency, which map elementary scene features to the probability of an observer focusing on those features, do not necessarily capture human gaze behavior in real-world scenes (6–8), though models combining bottom-up and top-down information have been developed, e.g. by adding task information as predictors in bottom-up models (9).

As an alternative to both top-down, investigator-defined, ROI-based approaches and bottom-up, computational approaches operating directly on scene characteristics, researchers have also explored using computational metrics of gaze dynamics as a way of comparing gaze patterns in a general and less assumption-laden fashion. These approaches examine differences in the spatiotemporal properties of looking points between individuals, without consideration or knowledge of underlying scene characteristics. However, many computational metrics for gaze behavior pattern comparisons explored in technical eye-tracking literature, such as string-edit-distance-based metrics (10–14), are not fully independent from ROIs. In addition, attentional heatmap-based metrics, although ROI-assumption free, contain no information regarding time sequence (15–17). Most importantly, metrics leveraging both spatial and temporal information (for example, spatiotemporal clustering (18,19), or vector-based scan paths (14), the attentional synchrony work of Smith and Henderson (20), or the multidimensional scaling distance matrix results of Nakano and colleagues (21)) have not been used to operationalize typical attention and/or to make group comparisons in autism research.

*Analytic Strategy*

*Normative Model.*

Step 1 aimed to define **Cohesion Values,** measures of similarity in gaze location during a given time frame between a TD toddler and all other TD toddlers. Towards this goal, gaze locations during the 3-minute video were broken down into contiguous “frames” lasting 200 ms each. For each frame, a median of the x and y gaze coordinates was calculated for each individual (median gaze locations), with the distance between median gaze locations of individuals *i* and *j* for frame *f* defined as $d_{i,j}^{f}$. We then calculated, for each individual, a representative distance to the TD group as a whole (taking care to never compare TD individuals against themselves) by taking the median of distances for each individual. Based on these values, for individual *i* and frame *f*, the Cohesion Value (${Cohesion}_{i,f}$) represented the similarity in gaze between individual *i* and all other TD toddlers:

${Cohesion}_{i,f}=\frac{100}{median\left( d_{i,j}^{f} \right)+\epsilon}, \forall j\in\{TD\},j\neq i$ (Equation 1)

with median pairwise distances $d_{i,j}^{f}$ calculated between individual *i* and all other toddlers *j* in the TD group. The value $\epsilon$ = 1 was added to avoid degeneracy in Cohesion Values when the median distance was 0.

In Step 2, we identified **High Cohesion Time Frames (HCTF)**. Conceptually, a HCTF represents a time interval when TD toddlers all focused their attention on the same areas of the screen. During HCTFs, TD toddlers had small median distances to each other; therefore the median Cohesion Value across all individuals in the group is high. HCTFs were computed by calculating the median Cohesion Value for each time frame across all individuals, i.e. median (*Cohesion_i,f_*, for all *i* $\in$ TD). The frames with the top 10% of median Cohesion Value across all frames were designated as HCTFs.

In Step 3, we defined **Typicality** scores as Cohesion Values during HCTFs, representing alignment with TD gaze behaviors. This allowed us to compare TD, DD and ASD toddlers to each other during these frames, using the aggregated performance of TD toddlers as a reference. Typicality scores were computed for each individual of each group for each condition (see Figure 2).

*ASD and DD cohesion models.*

To examine cohesive behaviors within each diagnostic group, we extended the normative model developed above to consider reference groups other than TD. This allowed us to ask what experimental conditions were associated with more organized behavior by toddlers with ASD and toddlers with DD. This aim involved the creation of **within-group cohesion models** for ASD and DD toddlers. Similarly to the model based on the TD group described above, the Cohesion values for each individual were computed as the inverted median distances from a given individual’s gaze to that of all individuals within their diagnostic group, e.g., the inverted median distance from one individual toddler with ASD to all ASD toddlers, and an individual DD toddler to all DD toddlers. In the normative model, the pairwise distances were always calculated by pairing a given individual with the TD toddlers’ gaze locations; whereas, in the within-group models, ASD toddlers were compared to all toddlers with ASD, and DD toddlers were compared to all DD toddlers. For the TD toddlers, the cohesion values in the normative and within-group models were the same.

Statistical analyses for this aim involved computing **proportions of high-cohesion time frames (P_HCTF_)** in within-group cohesion models *at an individual level*, reflecting the proportions of frames within each condition that were highly cohesive for each individual within his or her diagnostic group. Because, by definition, HCTFs represent the 10% of frames with the highest cohesion values, if the allocation of HCTFs across conditions is random, we would expect 10% of the frames within each condition to be marked as HCTFs. However, in practice the actual allocation of HCTFs across conditions deviated from 10%, either exceeding chance levels (*overexpression* of cohesion within a condition) or being lower than chance levels (*underexpression* of cohesion). Examining expression levels of cohesion in different conditions across the different diagnosis groups allowed us to identify commonalities in attentional salience that may be shared across members of particular groups (e.g., what draws attention most consistently in the ASD group). Formally, based on the within-group cohesion models modified from Aim 1a, Step 2, we allocated the HCTFs for all three groups respectively. For each individual, we counted the number of HCTFs within each condition and normalized it by dividing it by the total number frames in the condition (Equation 2):

$\mathrm{PHCTF}_{n}=\frac{C_{n}}{F_{n}} \times100$ (Equation 2)

Where C_n_ is the number of HCTFs within one of the four conditions, and F_n_ is the total number of time frames in that condition, where n ∈ {Sandwich, Dyadic Bid, Animated Toys}. The total number of frames were F_Sandwich_ = 315, F_Dydadic Bid_=346, and F_Animated Toys_ = 135.

*Results*

*Typicality Score Group Comparison.*

We computed the same diagnosis (3) x condition (3) linear mixed model on Typicality Scores with calibration accuracy as the covariate. The analysis indicated the same result: a diagnosis effect (*F*(2,307.6)=22.6, *p*<.001) and a diagnosis × condition interaction (*F*(4,614)=5.97, *p*<.001), but no condition effect (*F*(2,614)=2.1, *p*=.13). The calibration accuracy as a covariate was significant (F(1,307) = 147.4, p < 0.001).

We computed the same diagnosis (3) x condition (3) linear mixed model on Typicality Scores with percentage of valid looking as the covariate. The analysis indicated the same result: a diagnosis effect (*F*(2,307)=21.2, *p*<.001) and a diagnosis × condition interaction (*F*(4,610)=5.25, *p*<.001), but no condition effect (*F*(2,611)=2.1, *p*=.12). The percentage of valid looking was significant (F(1,737) = 128.7, p < 0.001).

We computed the same diagnosis (3) x condition (3) linear mixed model on Typicality Scores with Mullen nonverbal DQ as the covariate. The analysis indicated the same result: a diagnosis effect (*F*(2,302)=7.72, *p*=.001) and a diagnosis × condition interaction (*F*(4,605)=5.73, *p*<.001), but no condition effect (*F*(2,605)=2.03, *p*=.13). The Mullen nonverbal DQ as a covariate was significant (F(1,302) = 11.23, p = 0.001).

We computed the same diagnosis (3) x condition (3) linear mixed model on Typicality Scores with Mullen verbal DQ as the covariate. The analysis indicated the same result: a diagnosis effect (*F*(2,295)=6.91, *p*=.001) and a diagnosis × condition interaction (*F*(4,591)=5.92, *p*<.001), but no condition effect (*F*(2,591)=2.10, *p*=.12). The Mullen verbal DQ as a covariate was significant (F(1,295) = 5.12, p = 0.024).

*Correlation between Typicality scores and ROI looking time in within-group models*

In the main paper we analyzed the correlation between the Typicality score (in normative model) with ROI looking time percentages *across* all groups. In Table S1 we list the looking percentages at different ROIs in the cohesive frames of the normative model for each group and condition. In Table S2, we present the relationship of Cohesion to ROI looking percentages within each group. The results of the within group model follows the same patterns observed for the whole sample in the normative model. This suggests that while the proportion of high cohesive time frames (HCTFs) in each condition differs between groups, reflecting the likelihood of cohesive looking in the group in each condition (Main text, Figure 4), Cohesion variation *within* experimental conditions follows universal behaviors across groups: looking at the face in Dyadic Bid, looking at hands and the activity in Sandwich-making, and looking at Toys in the Animated Toys condition (Table S2).

Table S1. Percentage Looking (Mean (SD)) at predefined regions of interest (ROIs) in the Cohesive Frames of the Normative Model, stratified by experimental condition and diagnostic grouping.

| Condition | Group | %Valid | %Face | %Toys | %Body | %Table | %BG^1^ |
| --- | --- | --- | --- | --- | --- | --- | --- |
| Sandwich | ASD | 84 (17) | 9(10) | 5(8) | 6(9) | 78(19) | 3(4) |
|  | DD | 88(13) | 6(7) | 5(8) | 4(7) | 83(14) | 2(4) |
|  | TD | 88(16) | 6(7) | 4(6) | 3(5) | 85(14) | 2(4) |
| Speech | ASD | 74(21) | 66(22) | 12(12) | 4(6) | 9(11) | 6(7) |
|  | DD | 87(13) | 81(17) | 8(9) | 3(7) | 4(6) | 3(3) |
|  | TD | 82(19) | 81(16) | 6(7) | 4(9) | 4(6) | 4(7) |
| Toys | ASD | 83(21) | 4(10) | 86(19) | 1(3) | 1(4) | 8(15) |
|  | DD | 90(15) | 6(10) | 86(20) | 1(4) | 1(2) | 6(11) |
|  | TD | 86(20) | 2(4) | 91(15) | 1(3) | 0(2) | 5(10) |

^1^BG = Background

Table S2. Correlation between cohesion values and the percentage looking at predefined regions of interest (ROIs) in the Cohesive Frames of the Within-group Models, stratified by experimental condition. Dominant positive correlations are bolded.

| ASD = 111 |  |  |  |  |  |  |
| --- | --- | --- | --- | --- | --- | --- |
|  | Looking Percentage on ROIs | Face | Toys | Body | Table | BG |
|  | SW Cohesive Scores | -.43*** | -.63*** | -.33*** | **.77***** | -.64*** |
|  | DB Cohesive Scores | **.79***** | -.55*** | -.39*** | -.45*** | -.36*** |
|  | Toy Cohesive Scores | -.21* | **.72***** | -.33*** | -.23* | -.63*** |
|  |  |  |  |  |  |  |
| DD=36 |  |  |  |  |  |  |
|  | Looking Percentage on ROIs | Face | Toys | Body | Table | BG |
|  | SW Cohesive Scores | -.47*** | -.31 | -.32 | **.71***** | -.62*** |
|  | DB Cohesive Scores | **.62***** | -.39* | -.36* | -.36* | -.35* |
|  | Toy Cohesive Scores | -.13 | **.38*** | -.26 | -.37* | -.42* |
|  |  |  |  |  |  |  |
| TD=161 |  |  |  |  |  |  |
|  | Looking Percentage on ROIs | Face | Toys | Body | Table | BG |
|  | SW Cohesive Scores | -.50*** | -.36*** | -.55*** | **.73***** | -.44*** |
|  | DB Cohesive Scores | **.59***** | -.25** | -.33*** | -.32*** | -.44*** |
|  | Toy Cohesive Scores | -.16* | **.58***** | -.18* | -.27** | -.48*** |

* p<.05

** p<.01

***p<.001

*Proportion of frames with highest Cohesion Scores.*

Within-group linear mixed models were run to examine potential differences in the proportion of HCTFs allocated to each condition within each group. Effect sizes between conditions within each group are listed in Table S3.

Table S3. Between condition effect sizes (Cohen's *d*) for proportion of HCTFs.

| **Cohen’s d** | **ASD** | **DD** | **TD** |
| --- | --- | --- | --- |
| **SW - DB** | **4.28** | **-3.63** | **0.59** |
| **SW - Toy** | **-0.09** | **6.08** | **3.74** |
| **DB - Toy** | **-3.01** | **9.37** | **3.45** |

*Discussion for the within group dependency with the TD as the reference group*

For the Normative model, TD participants are used as a reference for other TD individuals. Though self-distances are excluded in the Cohesion value formulation (see Equation 1, Supplement), there is a small overlap in terms associated with the pairwise distance calculations. For instance, if there were only three individuals (1,2,3) in the TD group, given d(1,2)=d(2,1), there is a symmetric dependency in cohesion(1) and cohesion(2). However, we have large number of participants, this dependency is small in practice as the overlap between any two individuals is at most one pairwise distance (out of N-1 pairs, where N is the number of participants).

To further bound this effect, we conducted a simulation study where we randomly divided the TD group into two groups: a “test group (group A)” and a “reference group (group B)”. For each individual in group A the pairwise distances between A and every member of group B were calculated (but never between members of A and A, or B and B). The lowest 10% of distances were used as a cutoff for HCTFs. As expected, the mean score of the 10% across 1000 simulation studies was 50.08 (SD=.25), very close to the mean score of the full sample without splitting into two groups (M=49.79). The coefficient of variation due to splitting was .25/50.08 = .0050. By comparison, the data used to generate Table 2 indicated that the coefficient of variation across individuals was std(TD)/mean(TD) = 5.61/45.7  = .123, suggesting that the variation due to splitting was approximately 4% of inter-subject variation (.005/.123).

It is important to note that the coefficient of variation of the split group simulation is likely more affected by the decreased sample size due to splitting than it is by the (less than 1% overlap) in terms between and two individuals within the TD group.

*Discussion for Proportion of High Cohesion Time Frames:*

It is important to note that the high proportion of HCTFs in Animated Toys for the ASD group model does not represent an absolute higher level of cohesion. We defined HCTFs as the top 10% most cohesive time frames; therefore, the HCTFs were not identified by their absolute cohesive value, but rather each frame’s relative ranking. For example, the TD and DD groups may be similarly cohesive in the Animated Toys condition compared to the ASD group, but the Typicality score may be lower due to the increased draw for social information (overexpression in the Dyadic Bid and Sandwich conditions). Similarly, a high proportion of HCTFs in the Animated Toys condition in the ASD-group model could imply underexpression of cohesion in the Dyadic Bid or Sandwich conditions and not necessarily an increased cohesion during Animated Toys relative to the TD or DD groups. These results must be considered in the context of group variability.

High cohesion is formed by synchronized in-phase looking. For TD participants, TD cohesion scores (i.e. Typicality scores) are highest when they are presented with the DB condition, as is shown in Figure S1. Directionally, joint attention may direct the attention of some children from faces to objects. However, there is no guarantee that the children will look "in lockstep" with one another, i.e. that the direction of the gaze will occur on the same points at the same time, as is illustrated by Figure S2. In this figure we can see that even TD children are split between looking at the actress and looking at the toy to which she is gazing. In addition, significant eye movement between these two locations further exacerbates the identification of HCTFs. In our work we used relative ranking of cohesion scores, so what we see distinguish scenes that are highly cohesive than those weakly cohesive ones. Therefore joint attention may not be the most attention gathering time frame comparing to dyadic bid, sandwich or animated toys.

Figure S1. Screenshot of one high-cohesion time frame occurring during the Dyadic Speech condition. Green dots: TD toddler gaze locations.


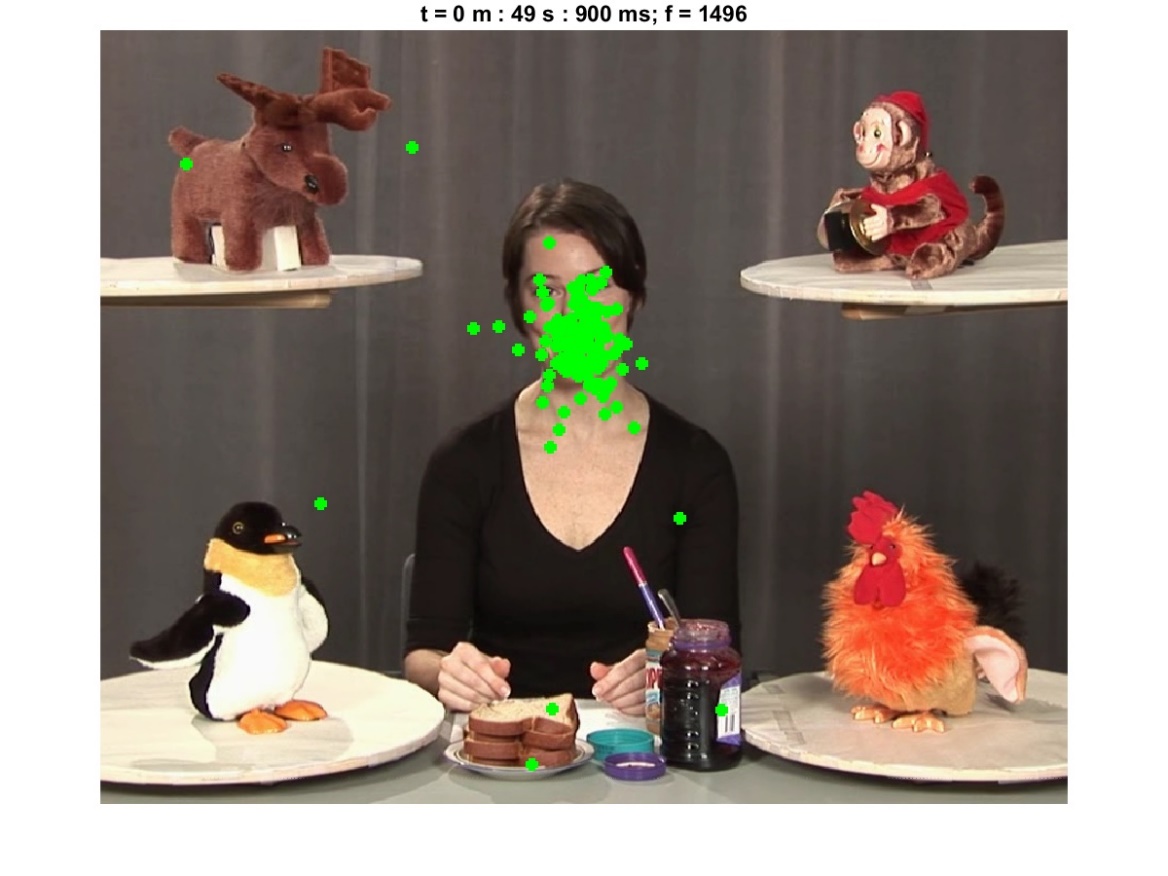


Figure S22. Screenshot of participants’ gaze position during a frame selected from the Joint attention condition. Green dots: TD toddler gaze locations.


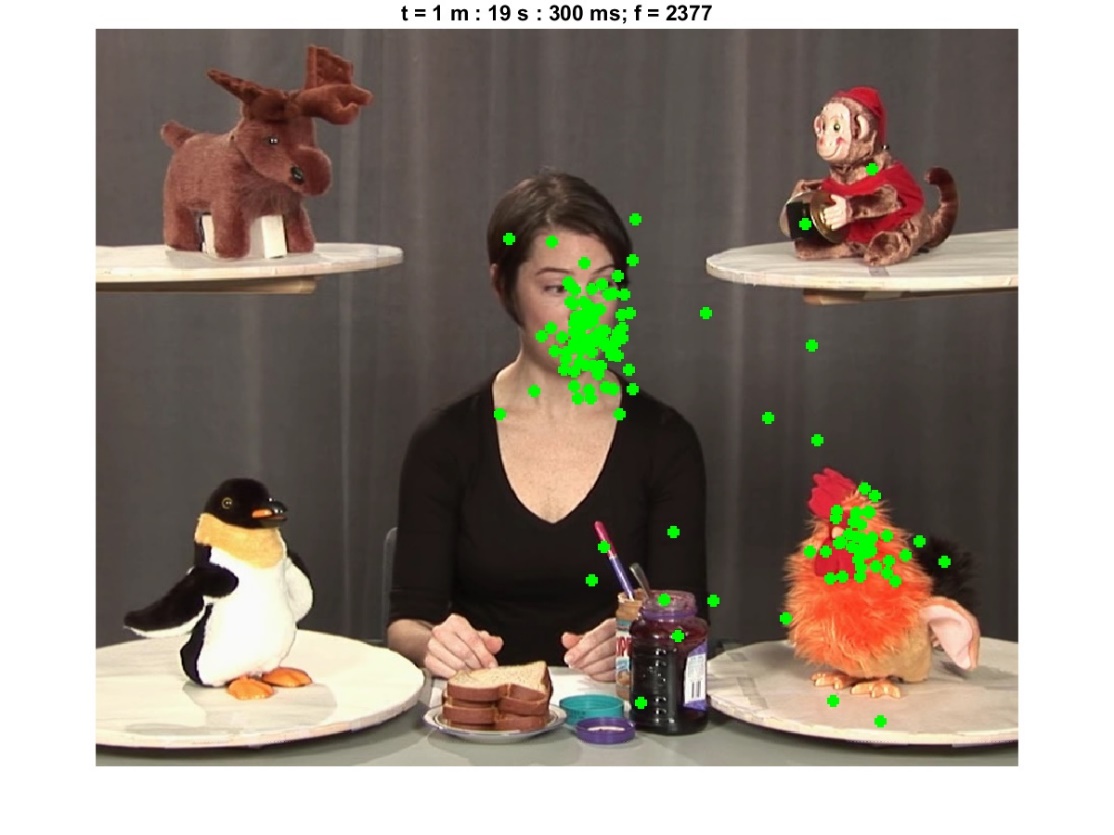


1. Itti L. Quantifying the contribution of low-level saliency to human eye movements in dynamic scenes. Vis Cogn. 2005;12(6):1093–123.

2. Sugano Y, Matsushita Y, Sato Y. Calibration-free gaze sensing using saliency maps. In: Computer Vision and Pattern Recognition (CVPR), 2010 IEEE Conference on. IEEE; 2010. p. 2667–74.

3. Wang S, Jiang M, Duchesne XM, Laugeson EA, Kennedy DP, Adolphs R, et al. Atypical Visual Saliency in Autism Spectrum Disorder Quantified through Model-Based Eye Tracking. Neuron. 2015 Nov 4;88(3):604–16.

4. Amso D, Haas S, Tenenbaum E, Markant J, Sheinkopf SJ. Bottom-Up Attention Orienting in Young Children with Autism. J Autism Dev Disord. 2013 Sep 8;1–10.

5. Shic F, Chawarska K, Lin D, Scassellati B. Measuring context: The gaze patterns of children with autism evaluated from the bottom-up. In: Development and Learning, 2007 ICDL IEEE 6th International Conference on. 2007. p. 70–5.

6. Ouerhani N, von Wartburg R, Hugli H, Muri R. Empirical validation of the saliency-based model of visual attention. Electron Lett Comput Vis Image Anal. 2004;3(1):13–24.

7. Shic F, Scassellati B. A Behavioral Analysis of Computational Models of Visual Attention. Int J Comput Vis. 2007 Jun 25;73(2):159–77.

8. Henderson JM, Brockmole JR, Castelhano MS, Mack ML. Visual saliency does not account for eye movements during visual search in real-world scenes. Eye Mov Window Mind Brain. 2007;537562.

9. Peters RJ, Itti L. Beyond bottom-up: Incorporating task-dependent influences into a computational model of spatial attention. In: Computer Vision and Pattern Recognition, 2007 CVPR’07 IEEE Conference on. IEEE; 2007. p. 1–8.

10. Levenshtein VI. Binary Codes Capable of Correcting Deletions, Insertions and Reversals. In: Soviet Physics Doklady. 1966. p. 707.

11. West JM, Haake AR, Rozanski EP, Karn KS. eyePatterns: software for identifying patterns and similarities across fixation sequences. In ACM Press; 2006 [cited 2017 Feb 10]. p. 149. Available from: http://portal.acm.org/citation.cfm?doid=1117309.1117360

12. Cristino F, Mathôt S, Theeuwes J, Gilchrist ID. ScanMatch: A novel method for comparing fixation sequences. Behav Res Methods. 2010 Aug;42(3):692–700.

13. Choi YS, Mosley AD, Stark LW. “ Starkfest” Vision and Clinic Science Special Issue: String Editing Analysis of Human Visual Search. Optom Vis Sci. 1995;72(7):439–51.

14. Jarodzka H, Holmqvist K, Nyström M. A vector-based, multidimensional scanpath similarity measure. In: Proceedings of the 2010 Symposium on Eye-Tracking Research & Applications. ACM; 2010. p. 211–8.

15. Reeder RW, Pirolli P, Card SK. Webeyemapper and weblogger: Tools for analyzing eye tracking data collected in web-use studies. In: CHI’01 extended abstracts on Human factors in computing systems. ACM; 2001. p. 19–20.

16. Ackland S, Istance H, Coupland S, Vickers S. An Investigation into Determining Head Pose for Gaze Estimation on Unmodified Mobile Devices. In: Proceedings of the Symposium on Eye Tracking Research and Applications [Internet]. New York, NY, USA: ACM; 2014 [cited 2015 Feb 15]. p. 203–206. Available from: http://doi.acm.org/10.1145/2578153.2578184

17. Bojko AA. Informative or misleading? Heatmaps deconstructed. In: International Conference on Human-Computer Interaction. Springer; 2009. p. 30–9.

18. Blascheck T, Kurzhals K, Raschke M, Burch M, Weiskopf D, Ertl T. State-of-the-art of visualization for eye tracking data. In: Proceedings of EuroVis. 2014.

19. Kurzhals K, Weiskopf D. Space-time visual analytics of eye-tracking data for dynamic stimuli. IEEE Trans Vis Comput Graph. 2013;19(12):2129–38.

20. Smith T, Henderson J. Attentional synchrony in static and dynamic scenes. J Vis. 2008;8(6):773–773.

21. Nakano T, Tanaka K, Endo Y, Yamane Y, Yamamoto T, Nakano Y, et al. Atypical gaze patterns in children and adults with autism spectrum disorders dissociated from developmental changes in gaze behaviour. Proc R Soc B Biol Sci. 2010 Oct 7;277(1696):2935–43.
